# Spatial differentiation and influencing factors of red tourism resources transformation efficiency in China based on RMP-IO analysis

**DOI:** 10.1038/s41598-024-61021-6

**Published:** 2024-05-10

**Authors:** Zhenjie Liao, Lang Wang

**Affiliations:** 1 School of Management, Guangzhou Huashang College, Guangzhou, China; 2 School of Economics and Management, Xianyang Normal University, Xianyang, China

**Keywords:** Red tourism, Tourism resource transformation efficiency, Modified DEA model, Interaction influence, China, Ecology, Environmental social sciences

## Abstract

This study employs modified data envelopment analysis (DEA) models and spatial autocorrelation methods to analyze the characteristics of red tourism transformation efficiency and categorize them into efficiency zones. By utilizing geographic detector models, the interactive driving mechanisms behind spatial differentiation are revealed, providing valuable insights for the high-quality transformation and development of China's red tourism economy. The application of modified DEA models facilitates the evaluation of red tourism resource transformation efficiency by decomposing comprehensive efficiency into single-factor efficiency for individual input and output variables. The results indicate that: (1) Expansion of tourism factors is crucial for achieving red tourism resource transformation in China, with low efficiency in resource endowment investment acting as the primary constraint. (2) Local spatial correlation between production efficiency and resource transformation efficiency demonstrates a decreasing trend from east to west, leading to the classification of China’s red tourism resources into five types of efficiency zones. (3) Endogenous ability factors predominantly affect red tourism resource transformation efficiency, with interaction between internal and external factors driving spatial differentiation.

## Introduction

With the sustained and rapid development of the Chinese economy and the rapid increase in resident income levels, tourism in China has experienced significant growth in both tourist numbers and income. It has established itself as a vital industry in the national economy and one of the fastest-growing consumer sectors^1^. However, tourism resources often exhibit fragility and scarcity, and the expansion of the tourism industry can pose threats to the surrounding environment^2^. Sustainable development of the tourism industry necessitates a harmonious coexistence between humans and nature to promote ecological balance and long-term viability^3,4^. Realizing sustainable development within the regional tourism industry drives a shift in development paradigms toward low-carbon economic development^5^ and ensures alignment between economic, environmental, and social benefits^6^. As human economic activity continues to expand, the ability to exploit nature grows; however, the resilience of the natural environment diminishes^7^. Consequently, various countries recognize the interdependence between tourism economic development and ecological environmental protection^8^, making the quality of tourism-driven growth and development modes a focal point for scholars worldwide. Red tourism, a significant component of China’s tourism economy, serves as both a key political and cultural initiative and an important economic project^9^. Effective utilization and management of red tourism resources are significant for the preservation and propagation of revolutionary heritage and a cornerstone for advancing the new “double-cycle” development strategy^10^.

## Literature review

The concept of “efficiency” was proposed by British economist Farrell in 1957 and widely applied in various disciplines such as economics and management^11^. In tourism research, researchers worldwide primarily focused on the operational and management efficiency of hotels and restaurants^12,13^. As research progressed, the scope expanded to encompass areas such as travel agencies, tourism transportation, scenic spots, and tourism ecology^14–17^. Presently, comprehensive evaluation of regional tourism industry development efficiency has emerged as a prominent topic in academic research^18^, particularly focusing on regional differences and influencing factors impacting total factor production efficiency in the tourism industry^19–22^. Tourism efficiency denotes the effective input to the output of tourism resources at a certain level of production technology^23,24^. It serves as a comprehensive reflection of the effective allocation, operational status, and management level of tourism input factor resources^25,26^.

Research on tourism efficiency is pivotal in destinations in discovering ineffective utilization of tourism-related resources, optimizing resource allocation structures, and promoting high-quality development of the tourism economy. The allocation of tourism resources, is also a type of resource allocation and scheduling problems, can be addressed using traditional scheduling theory and optimization algorithms to improve efficiency under different evaluation indicators^27–30^. Recently, numerous scholars have used the data envelopment analysis (DEA) model to evaluate tourism resource allocation efficiency, which considers multiple inputs and outputs^31,32^, offering a comprehensive evaluation of resource allocation efficiency in the tourism industry^33^. Liu et al.^34^ integrated energy consumption and carbon emissions into the analysis framework of green productivity in the tourism industry, measuring the green productivity of tourism in the Yangtze River region using a super-efficient DEA model. Avelino and Sasaki^35^ established the index sustainability assessment (SEMPAI) method within the DEA framework to evaluate marine protected areas, aligning environmental, socio-economic, and governance needs to enhance the sustainable development capacity of tourism marine protected areas, thereby improving management and long-term sustainability of coastal settlements in tourist destinations. Chaaboun explored spatiotemporal differences in inter-provincial tourism development in China, using the Bootstrap DEA model to evaluate tourism development levels in 30 provinces and cities, concluded that average tourism efficiency in the eastern region surpasses that in the central and western regions^36^. However, limited quantitative research exists on performance evaluation and resource allocation of tourism resource efficiency conversion efficiency from the perspective of sustainable development.

Therefore, this study builds upon previous research results^37^ and utilizes the existing modified DEA model to evaluate the resource transformation efficiency of 300 typical red tourism attractions in China in 2022. It analyzes the spatial variation characteristics in the transformation efficiency of red tourism resources using the spatial autocorrelation method, delineates transformation efficiency zones, and reveals the interactive driving mechanism of spatial variation in transformation efficiency with the geodetic detector model. Therefore, this study aims to address the shortcomings of the comprehensive output efficiency evaluation of input factors and provide scientific insights for the high-quality transformation and development of China's red tourism economy.

## Research methodology

### Index construction

#### Evaluation of the transformation efficiency of red tourism resources

Red tourism is an important application scenario of red culture across history, theory, and practice, serving as a platform for expressing its value and significance to society. In this field, red culture exhibits a unique attraction, theme, and sense of experience. Evolving alongside societal progress, the connotation and reach of red tourism resources continually adapt. These resources, as pivotal conduits for red tourism, embody both tangible reality and intangible spiritual initiative, holding profound social, mass, and historical significance. They encapsulate national and ethnic symbolism, reflecting a social construct intertwined with political, cultural, and tourism elements.

Efficiency in utilizing tourism resources includes total, technical, and scale efficiencies. Core to this theory is the concept of P-ness, focusing on the efficacy of transforming tourism products through innovation and spatial configuration. This framework integrates resource analysis (R-ness) and market analysis (M-ness), addressing tourism resource endowment, competitiveness, consumption patterns, and preferences of tourism subjects.

Therefore, drawing on the theoretical framework of resource-market-product (RMP), we construct a multi-input factor index for evaluating the transformation efficiency of red tourism resources: “resource endowment (resource)/tourism market (market)/product efficiency (product)-input/output” (RMP-IO). This evaluation system incorporates input factors such as red tourism market dynamics and tourism resource endowments, alongside output factors representing tourism product efficiency.

Based on existing research^37,38^ and following the principles of data accessibility and input indicator relevance, the RMP-IO framework select specific indicators: (1) Red tourism resource endowment input indicators. Key indicators include the value of tourism resources, environmental quality, and resource development conditions. Considering that grading China's red tourism resources poses challenges, input and maintenance costs are used as proxies for resource value. Environmental quality is approximated through the average ecological governance input index of attractions over the past 5 years^39^. Public and ancillary facilities serve as indicators for resource development^40^. (2) Red tourism market input indicators: Land, capital, and labor constitute fundamental production factors in the market economy. However, owing to challenges in defining and obtaining statistical data for tourism land, it is excluded from the evaluation methods^41^. Capital, crucial for tourism activities, is represented by the number of travel agencies and hotels, reflecting service and reception capacities respectively^27,28^. Given data limitations for quantifying the tourism labor force, the number of tour guide license holders serves as a proxy indicator^42^. (3) Red tourism product benefit output indicator: Social benefits take precedence in red tourism, emphasizing the unity of social, economic, and ecological benefits^43^. Tourism revenue and tourist trips quantify economic and social benefits respectively. Additionally, the attention garnered by red tourist attractions in modern media reflects social public opinion benefits^44–47^.

Local spatial autocorrelation examines the spatial characteristics of attribute values within geographic elements. Local Moran's *I*_*i*_ statistic is used to analyze the degree of local spatial dependence on the efficiency transformation of red tourism resources, with the calculation formula represented as follows:

1 $${I_i} = \frac{{\frac{{{X_i} - \overline X }}{{m_1}}\sum\limits_{j = 1}^n {{W_{ij}}({X_j} - \overline X )} }}{{\frac{{\sum\limits_{j = 1,i = 1}^n {{{({X_j} - \overline X )}^2}} }}{n}}}$$

2 $$S_X^2 = \frac{{\sum\nolimits_j {\left[ {{W_{ij}}{{({X_j} - \overline X )}^2}} \right]} }}{N}$$

where $S_X^2$ denotes the variance. The spatial patterns of red tourism resource transformation efficiency are categorized into five types based on local autocorrelation: HH cluster area (high-value cluster), LL cluster area (low-value cluster), HL cluster area (high and low-value cluster), LH cluster area (low and high-value cluster), and NN area (non-significant).

The study adopts a bivariate local spatial autocorrelation model to analyze the spatial correlation between the input and transformation efficiencies of China’s red tourism resources. It visualizes the correlation characteristics employing the following formula:

3 $$I_i^{KI} = \frac{{X_i^K - \overline {X_K} }}{{\sigma^K}}\sum\nolimits_{j = 1}^n {} \left[ {{W_{ij}} \times \frac{{X_i^I - \overline {X^I} }}{{\sigma^I}}} \right]$$

where ${I}_{i}^{KI}$ represents the bivariate local spatial autocorrelation coefficient of region *i*;${ X}_{i}^{K}$ denotes the value of the *K*th tourism resource input efficiency of region *i*; $X_j^{I}$ is the value of the *I*th tourism resource transformation efficiency of region *j*; $\overline{{X}_{K} }$ and $\overline{{X}^{I}}$ are the mean values of the *K*th and *I*th tourism resource efficiencies, respectively; and *σ*^*K*^ and *σ*^*I*^ are the variances of the *K*th and *I*th tourism resource efficiencies, respectively.

#### Analysis of influencing factors of spatial differentiation of transformation efficiency of red tourism resources

(1) Selection of factors influencing the transformation efficiency of red tourism resources

As an economic geographic factor, the evolution of spatial patterns in red tourism resource transformation efficiency undergoes a complex process affected by both internal and external factors. Internal factors serve as the core driving forces, while external factors act as catalysts, shaping spatial and temporal patterns through mutual influence. Accurately identifying and analyzing the dominant factors driving this change and their interactions is crucial for understanding the spatial differentiation of red tourism resource transformation efficiency. This analysis informs mechanisms and strategies to enhance quality and efficiency, fostering high-quality regional red tourism development.

Given the pivotal role of regional internal and external environmental differences in shaping spatial differentiation, ten factors are selected from the dimensions of endogenous capacity and external environment for analysis. These factors drawn from relevant research^37,48,49^, are selected based on data accessibility. The five endogenous ability factors encompass competitiveness, attractiveness, market scale, demand structure, and transformation mode of red tourism resources. The five external environment factors include location, transportation, economic development, industrial agglomeration, policy support, and informationization level of red tourism resources (Table 1).

**Table 1:** Effective factors and descriptive statistics of the transformation efficiency of red tourism resources.

Target class	Indicator category	Calculation method	Maximum values	Minimum value	VIF
Endogenous capacity	E1 Comparative interests of industries	Adoption of an indicator for the ratio of tourism output per capita to regional industry-wide output per capita/per cent	4.009	0.938	1.486
	E2 Product Innovation Capability	Adoption of fixed asset investment indicators for tourist attractions/billion dollars	417.221	29.433	4.619
	E3 Quality of human capital	Adoption of the indicator on the size of students in secondary vocational institutions and above / 10,000 persons	866,140	11,586	4.546
	E4 Tourism market size	Adoption of gross merchandise sales indicators/trillion yuan	21,291.42	25.83	2.775
	E5 Tourism industry structure	Adoption of Gross Tourism Receipts to GDP Indicator /%	21.301	0.655	1.716
	E6 Transportation network conditions	Adoption of time cost indicator/h for reaching attractions	2.656	0.105	2.496
External environment	E7 Level of economic development	Adoption of GDP per capita indicator/(million dollars per person)	164,889.5	35,994.81	8.912
	E8 Level of urbanization	Adoption of population urbanization rate indicator/%	89.3	35.73	4.632
	E9 Strength of policy support	Adoption of the indicator on the number of communications from government departments on red tourism/cases	498	14	4.463
	E10 Level of scientific and technical information	Adoption of the indicator for the ratio of regional postal traffic to total provincial postal traffic/%	18.8	0.3	1.529

(2) Interaction analysis of factors affecting the transformation efficiency of red tourism resources

Geo-detectors are used to analyze factors influencing the spatial heterogeneity of red tourism resource transformation efficiency and their interactions. The method, grounded in spatial differentiation and set theory, calculates the determining force index *Q*_*v*_ of regional differentiation by superposing factor forces spatially. It assesses the spatial differentiation of individual factors and the consistency of multifactor distributions, thus revealing interaction effects. The calculation formula is as follows:

4 $${Q_v} = 1 - \frac{1}{{n{\sigma^2}}}\sum\limits_{h = 1}^l {n_h} \sigma_h^2$$

where *n*_*h*_ is the number of samples within type *h* included in factor *A*_*i*_; *n* is the total number of samples in the study area; *l* is the number of classifications of factor *A*_*i*_; *σ*^2^ is the discrete variance of the study area; and ${ \theta }_{h}^{2}$ is the discrete variance of the sub-region. When ${\theta }_{h}^{2}$≠0, the model is valid and *Q*_*v*_ ∈ [0, 1]; the larger *Q*_*v*_ is, the greater the impact of factor *A*_*i*_ on resource transformation efficiency.

To assess the explanatory power of the interaction among multiple influencing factors on the spatial differentiation of regional red tourism resource transformation efficiency and reveal the mechanism of interaction occurrence, interaction detection is used. This method analyzes differences in strength, direction, linearity, or nonlinearity between multiple factors to determine whether their combined effect strengthens or weakens the explanatory power of the change of resource transformation efficiency. It also investigates whether the effects of the two factors on each other are interdependent. The interaction detection process is described in the literature^50^.

### Data sources and processing

#### Data sources

1. Red tourism resource data: input data come from China and provinces' red tourism development plans; output data come from the China Red Network (http://www.crt.com.cn/ybgjq.html), the Ministry of Culture and Tourism (https://redtourismmct.gov.cn/), and the official websites of each red tourist attraction.
2. Geospatial data: administrative division data from the Ministry of Civil Affairs (http://xzqh.mca.gov.cn/map); county-level vector boundary data from the National Center for Basic Geographic Information (http://dxzxmh.geo-compass.com); red tourist attraction location data from (online map.com) (http://www.gpsspg.com/maps.htm); traffic data from National Traffic Data Information and Statistics Network (http://tools.2345.com/jiaotong/lc.htm).
3. Social and economic statistics: 2021 China Statistical Yearbook, China Tourism Statistical Yearbook, China Urban Statistical Yearbook, China Statistical Yearbook.
4. Network statistics: In the era of big data, the Internet has become an important carrier for tourism information and recording tourism activities. Ma Fengwo and Ctrip Travel, popular travel itinerary-sharing websites in China, can efficiently and conveniently obtain comprehensive itinerary information from tourists. Network statistics are mainly from China Tourism (https://www.ct.cn), Ctrip (https://www.ctrip.com), Where to Go (https://www.qunar.com), and Hornet's Nest (https://www.mafengwo.cn).

#### Data processing

To ensure the coherence of the data, the interpolation method was used to supplement the missing values for the missing data in some provinces; taking into account the impact of the outliers of the impact factor data, the 10 factors were logarithmically transformed and then included in the model for calculation; taking into account the problems of the multi-source of the impact factor data, the error, endogeneity and covariance between the data, the missing values and outliers were checked, corrected and eliminated in the process of the data pre-processing. Based on the data preprocessing process, Cronbach’s α coefficient method (> 75%) and variance inflation factor (> 10) of SPSS software were used to test the reliability of the batch of data. In the process of data processing, while ensuring that it is conducive to result analysis, the influence of subjective factors should be minimized as much as possible, and relevant indicators should be flattened. At the same time, this article logarithmically processed the relevant indicators to reduce heteroscedasticity caused by data volatility. Using SPSS software, the Variance Inflation Factor (VIF) index was used to test whether there was multicollinearity among these 10 independent variables. The results showed that all variables had VIFs between 1 and 10, indicating that the problem of multicollinearity among the explanatory variables was not serious and could be further analyzed.

### Ethical approval

This article does not contain any studies with human participants performed by any of the authors.

## Analysis of results

### Evaluation results of red tourism resource efficiency in China

#### Evaluation of input–output efficiency of red tourism resources in China

**Figure 1:**
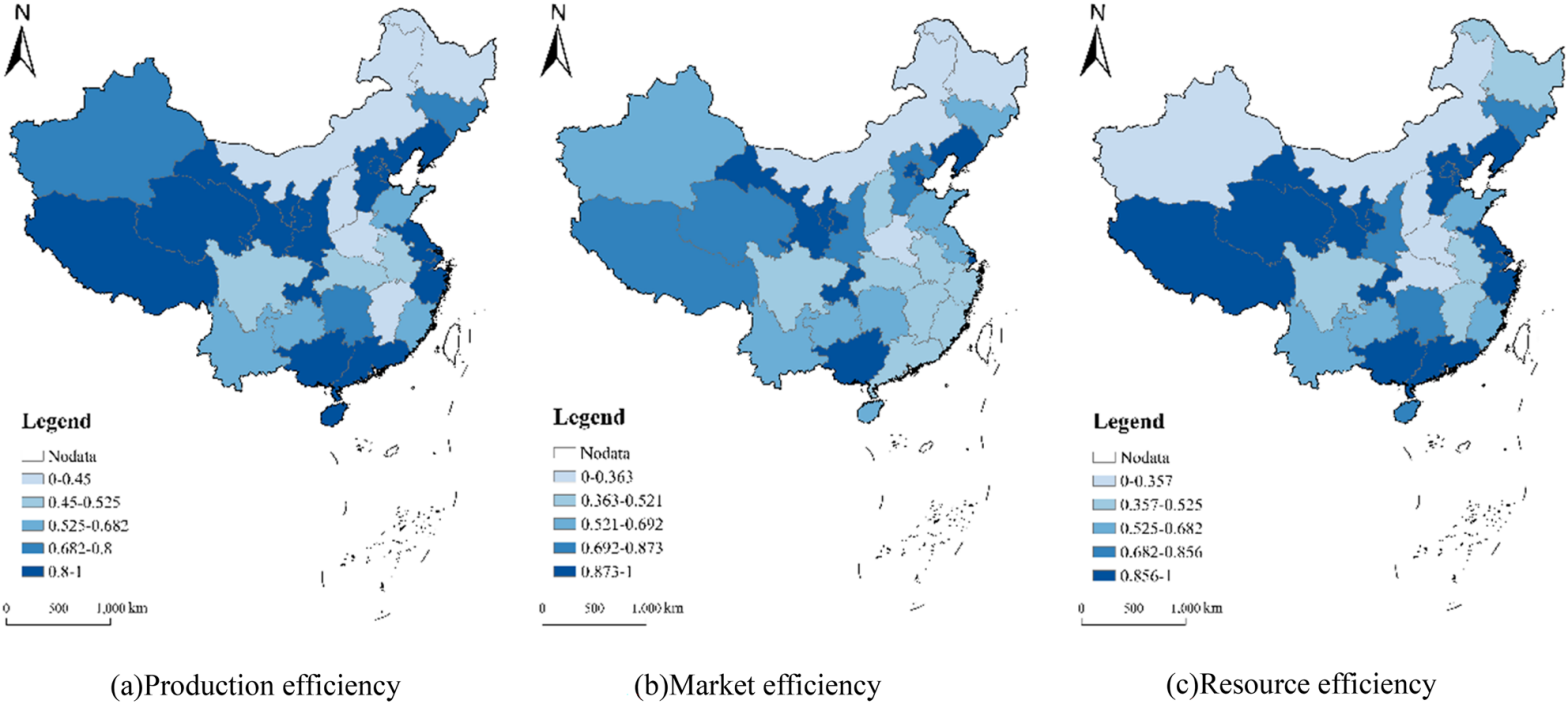
Spatial distribution of red tourism resource efficiency in China in 2022.

Equations (1) and (2) were used to evaluate the efficiency of 300 red tourism resources commissioned in China in 2022. These resources were categorized using the ArcGIS natural breakpoint method, yielding the mean efficiency values by province (Fig. 1).

(1) China's red tourism resources commissioning efficiency deviates from the frontier surface, showing the spatial characteristics of gradual decrease from east to west. In 2022, the average commissioning of China's red tourism resources stood at 0.72. Notably, the commissioning, market, and resource endowment efficiencies vary significantly among different resource units and provinces, revealing an overall spatial trend of gradual decline from east to west. The evaluation results of resource units show that 12 of the 300 red tourism resource evaluation units are at the forefront of production (production efficiency is 1) (Fig. 1a). The market efficiency of 34 red tourism evaluation units is at the production frontier (Fig. 1b), and there are 25 red tourism resource endowment efficiency at the forefront (Fig. 1c).

(2) Decomposing the efficiency of China's red tourism resources regarding input and output reveals the allocation capacity and technical innovation status of these resources. The mean pure technical efficiency of China's red tourism resources is 0.611, demonstrating a spatial trend of Y. Evaluation results indicate that while the seven evaluation units at the production frontier maintain the highest pure technical efficiency, the unit with the lowest efficiency value changes, notably the former residence of Saifudin Aiz in Artux City, Kizilsu Kyrgyz Autonomous Prefecture, Xinjiang Uygur Autonomous Region. Moreover, the pure technical efficiency of the 31 provinces demonstrates a spatial trend decreasing from east to west. The average pure technical efficiency of tourism resources in the east stands at 0.582, representing a 12.98% reduction from total production efficiency. Conversely, the pure technical efficiency of tourism resources in the West is the lowest at 0.254, reflecting the largest reduction (27.08%) compared to total production efficiency. Other regions similarly demonstrate smaller pure technical efficiencies, all exhibiting different degrees of decline relative to total production efficiency. These results underscore the low technical innovation capacity and the inadequate allocation of tourism resources as significant constraints on the improvement of production efficiency within China’s red tourism sector.

#### Results of the transformation efficiency of China's red tourism resources

**Figure 2:**
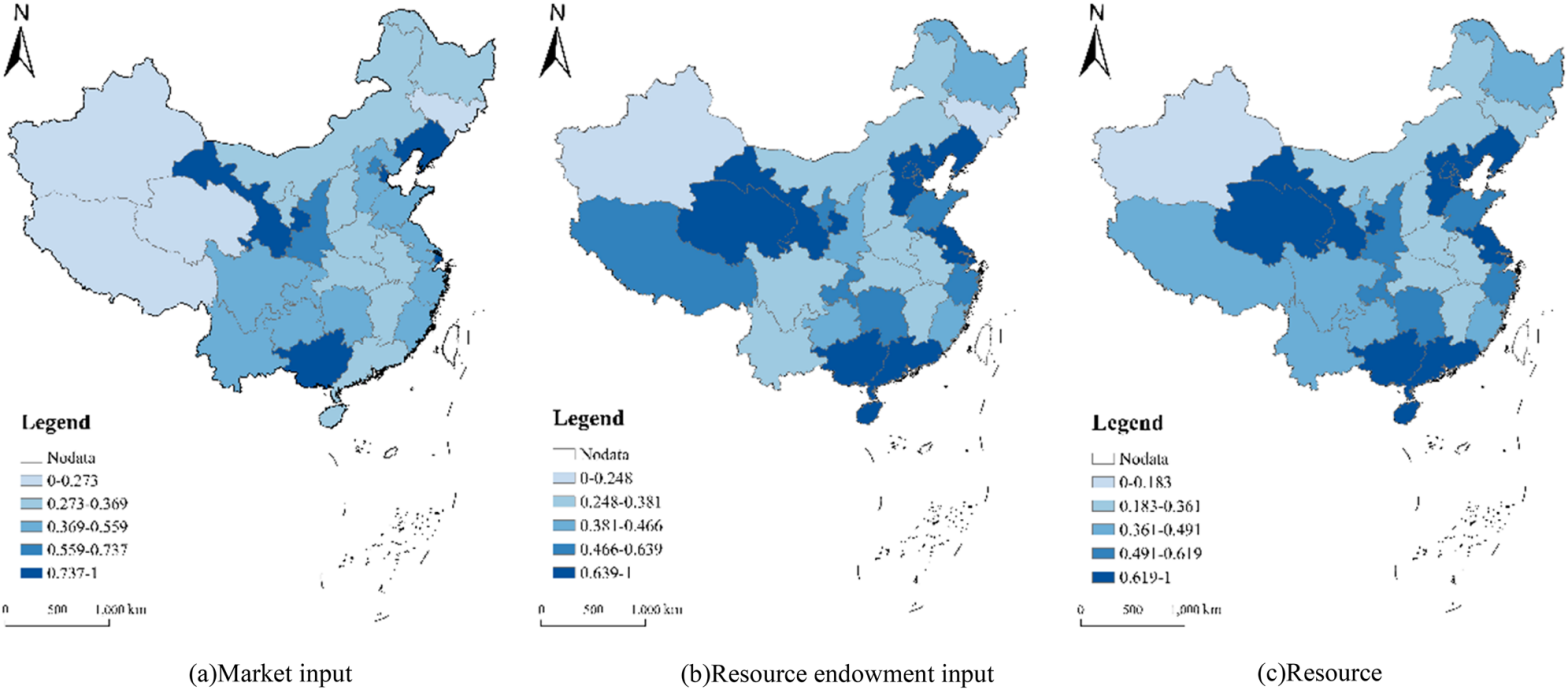
Spatial distribution of red tourism resource transformation efficiency in China in 2022.

The evaluation results of market input conversion efficiency show that the average value of market input conversion efficiency of China's red tourism market in 2022 is 0.563, which is at a medium–low level, and the input conversion efficiencies of the number of travel agencies, the number of tourism employees and the number of hotels show a low-value equilibrium structure (Fig. 2a). The evaluation results of resource endowment input conversion efficiency show that the average conversion efficiency of China's red tourism resource endowment input in 2022 is 0.573, which is at a low level, and the input conversion efficiency of factor value, environmental quality and resource development presents a low and unbalanced structure. However, the conversion efficiency of resource endowment input at the regional level presents a banded pattern of “flower arrangement” with high and low values (Fig. 2b).

China's red tourism resources conversion efficiency lags behind the frontier, showing a “V-shaped” spatial pattern of local high values. 2022 China's red tourism resources conversion efficiency average value is 0.549, the economic effect of the conversion efficiency (0.340) has become the main factor affecting the conversion efficiency of China's red tourism resources. Regionally, the resource conversion efficiency as a whole shows a spatial difference of gradual change from east to west (Fig. 2c). The mean value of conversion efficiency is higher in the eastern provinces, and the high conversion efficiency of the social effect of tourism resources has become a factor driving the efficiency of conversion in the region; while the important factor limiting the region is the relatively low conversion efficiency of the economic effect, with the most prominent performance being in Beijing (1).

The developed transportation network in the east, coupled with the robust tourism consumption demand from rapid economic development, enhances conversion efficiency to varying degrees. The conversion efficiency of the western provinces is the highest at 1, and Qinghai Province is the highest area in China (1). The region's red tourism resource conversion efficiency is characterized by high conversion efficiency of social effects and extremely low conversion efficiency. This structural pattern arises from the influence of the region’s rich geographic landscape tourism resources, which significantly improve the radiation and driving effect of red tourism, thereby improving social effect conversion efficiency. However, this influence does not extend to economic effect conversion efficiency, resulting in its pronounced disparity.

Under the influence of the “siphon effect” of the resources of architectural facilities and geographic landscape tourism, the flow preference of the market main body and the concentration of social concern have led to the lower conversion efficiency of economic benefits and public opinion effects. The conversion efficiency of the western region is 0.407 lower than that of the eastern and southern regions, but its composition of conversion efficiency is different, that is, the conversion efficiency of social effects is high but the conversion efficiency of public opinion effects is low. Owing to the imperfect red tourism market in the western region, imperfect transportation network conditions, and inadequate promotion of red tourism, the spillover effects of tourists' “words and deeds” and tourism enterprises' “advanced experience” in neighboring areas are insufficient, hindering the transformation of public opinion benefits.

### Geographical exploration results of spatial differentiation of red tourism resources transformation efficiency in China

#### Impact factor test

To accurately identify the influencing factors affecting the spatial differentiation of China's red tourism resources’ transformation efficiency, a logistic regression model was used to analyze the regression coefficients and multivariate covariance of the ten indicators (Table 2). The regression results indicate a significant positive correlation (p < 0.01) between the quality of human capital (E3), tourism market scale (E4), tourism industry scale (E5), urbanization level (E8), policy support strength (E9), and the spatial differentiation. Additionally, covariance diagnosis reveals a positive correlation among industry comparative interests (E1), product innovation ability (E2), transportation network conditions (E6), economic development level (E7), and scientific and technological information level (E10). However, factors (E7) and (E10) were excluded because of variance expansion coefficients exceeding 10. To validate the scientific validity of retained and excluded factors, the natural breakpoint method was used for classification, followed by spatial coupling and matching analysis with tourism resource transformation efficiency classes. The results revealed a high degree of overlap between factor types E3, E4, E5, E8, and E9 with class 0 (perfect match class), thus identifying these five factors as dominant factors affecting the spatial differentiation of red tourism resource transformation efficiency.

**Table 2:** The regression coefficients and multivariate covariance of the ten indicators.

Variable	Coefficient	VIF	Confidence degree
E1	0.06161	11.235	
E2	0.01131	15.69	
E3	0.01119	0.79663	***
E4	0.09733	0.3184	***
E5	0.01908	0.110803	***
E6	0.09365	30.862	
E7	0.00371	29.624	
E8	0.02498	0.86245	***
E9	0.04522	0.53833	***
E10	0.07235	15.385	

***, **, and * represent significant at 1%, 5%, and 10% significance levels, respectively.

#### Influence detection results for dominant influence factors

To assess the influence of the influencing factors on the transformation efficiency of regional red tourism resources, geo-detectors were used to detect the 300 red tourism sites (transformation efficiency values) and their distribution areas (transformation efficiency mean values), and the following results were obtained: (1) The influence of the dominant influencing factors varied significantly, and the comprehensive influence of the external environment became the main source of influence. The dominant factors affecting the transformation efficiency of China's red tourism resources are E5, E8, E9, E3, and E4 in order at the 5% significance level, and their corresponding influences are 0.3565, 0.3237, 0.3132, 0.2458 and 0.1761, respectively. The combined influence of the external environment is 0.6369, and the combined influence of endogenous capacity is 0.7784, which is 0.1415 bigger than the influence of the external environment (Table 2).

The spatial differentiation of the influence of the dominant factors is obvious, showing a Y-shaped spatial pattern. In the provincial dimension, the combined influence of the red tourism resource transformation efficiency factor in Beijing, Tianjin, Gansu, Ningxia, Guangdong, and Hainan is at a high level. From the geographical partition dimension, the mean value of the integrated influence of the factors in the eastern region of China is 0.2653, forming a high-influence extreme zone, while the mean value of the low-influence extreme zone in the northwest is 0.0792. From the transformation efficiency partition dimension, the integrated influence of the factors in the synergistic zone of the tourism resources input and output efficiency is the largest at 0.4098, and the integrated influence in the zone of the transformation and upgrading of the efficiency of the tourism resources is the smallest at 0.2626.

#### Interaction detection results for dominant influence factors

(1) The interaction influence of two-factor is stronger than that of one-factor and dominated by nonlinearly enhanced interaction type. The factor interaction influences of the five types of regions of China's red tourism resource transformation efficiency all show nonlinear enhancement and two-factor enhancement, and there is no independent and weakened relationship. Among them, E2, E9, E7, and E4 influence factor groups have high mean values of interaction influence compared to single-factor influence, and become the strongest factor groups influencing the transformation efficiency of China's red tourism resources. In the overall analysis, among the factor interaction types in the region, the nonlinear enhancement category is 51, accounting for 86.67% of the total number of groups, and the two-factor enhancement category accounts for 13.33% of the total number of groups. Analyzing the internal and external influence factors, the internal and external combined nonlinear increase interaction types accounted for 66.67% of the total number of groups, while the intra-group interactions were dominated by bifactor enhancement accounting for 53.33% of the total number of groups. The results show that the interaction of two factors have a more profound impact on the transformation efficiency of China's red tourism resources, and the cyclic cumulative effect of the combined influence of internal and external factors more realistically depicts the formation of its spatial differentiation pattern, and it becomes the key to the future enhancement of the transformation efficiency of China's red tourism resources and the high-quality development of red tourism.

(2) The two-factor interaction influence showed differences in internal and external types, and the cumulative cyclic effect of the endogenous ability factor group was more significant. Endogenous capacity factors become the dominant factors affecting the conversion efficiency of red tourism resources. The average interactive influence of influencing factors on the transformation efficiency of China's red tourism resources in 2022 is successively E2, E3, E4, E9, E6, and E7. Compared with the single factor influence, this result shows a big difference, that is, product innovation ability, human capital quality, tourism market scale, policy influence, and influence status increase. Among them, the factor with the biggest improvement is product innovation ability; The influence status of external environmental factors declined, and the most significant factor was traffic network conditions. Therefore, paying attention to the interaction and coupling of endogenous capacity influencing factors of red tourism resource conversion efficiency and promoting the integration and linkage of multiple policies without losing an opportunity provides a new direction for systematically improving the conversion efficiency. The interaction of internal and external factors is the main way of spatial differentiation of red tourism resource conversion efficiency. The most significant interaction effects of the external environment and endogenous capability factors are E2 ∩ E9, E3 ∩ E4, and E3 ∩ E7, whose average influence on the transformation efficiency of China's red tourism resources is as high as 0.5864, 0.5712, and 0.5608. The external environmental factors of transportation network conditions, economic development level, and policy support are the basis for the development of red tourism resources and the prerequisite for changes in transformation efficiency; The three endogenous factors of product innovation capability, human capital quality, and tourism market size serve as the core competitiveness of the development of red tourism resources and are the driving force for changes in transformation efficiency. Therefore, focusing on the linkage between internal and external factors provides a new way to improve the efficiency of transforming red tourism resources (Table 3).

**Table 3:** Influence of dominant factors of the transformation efficiency of red tourism resources in China in 2022.

Region	E3	E4	E5	E8	E9
Beijing	0.0231	0.0412	0.0501	0.0687	0.0904
Tianjin	0.0792	0.0321	0.0783	0.0452	0.0820
Hebei	0.0392	0.0517	0.0178	0.0623	0.0729
Shanxi	0.0283	0.0913	0.0608	0.0541	0.0375
Inner Mongolia	0.0782	0.0356	0.0852	0.0423	0.0184
Liaoning	0.0514	0.0654	0.0346	0.0625	0.0973
Jilin	0.0764	0.0539	0.0593	0.0768	0.0231
Heilongjiang	0.0673	0.0381	0.0394	0.0895	0.0839
Shanghai	0.0823	0.0756	0.0498	0.0452	0.0892
Jiangsu	0.0612	0.0392	0.0876	0.0352	0.0562
Zhejiang	0.0423	0.0738	0.0293	0.0712	0.0831
Anhui	0.0487	0.0657	0.0219	0.0538	0.0472
Fujian	0.0784	0.0619	0.0857	0.0391	0.0613
Jiangxi	0.0354	0.0927	0.0348	0.0475	0.0792
Shandong	0.0695	0.0392	0.0734	0.0629	0.0873
Henan	0.0453	0.0526	0.0625	0.0831	0.0384
Hubei	0.0735	0.0274	0.0287	0.0764	0.0572
Hunan	0.0543	0.0652	0.0876	0.0395	0.0693
Guangdong	0.0632	0.0821	0.0452	0.0518	0.0492
Guangxi	0.0482	0.0582	0.0712	0.0892	0.0653
Hainan	0.0392	0.0634	0.0812	0.0532	0.0792
Chongqing	0.0572	0.0398	0.0873	0.0492	0.0627
Sichuan	0.0631	0.0742	0.0539	0.0476	0.0839
Guizhou	0.0493	0.0312	0.0762	0.0378	0.0926
Yunnan	0.0542	0.0643	0.0485	0.0672	0.0782
Tibet	0.0752	0.0287	0.0598	0.0452	0.0378
Shaanxi	0.0493	0.0527	0.0738	0.0395	0.0682
Gansu	0.0623	0.0382	0.0729	0.0875	0.0512
Qinghai	0.0823	0.0497	0.0391	0.0743	0.0286
Ningxia	0.0762	0.0537	0.0276	0.0487	0.0792
Xinjiang	0.0673	0.0374	0.0812	0.0578	0.0396
Production efficiency synergy zone	0.4080	0.4043	0.3722	0.4089	0.3026
Improving quality and efficiency zone	0.3900	0.2997	0.397	0.3000	0.4413
Upgrading efficiency conversion zone	0.2728	0.2709	0.3515	0.2625	0.3708
Improvement production capacity zone	0.2915	0.2755	0.2523	0.3071	0.2455
Effectiveness cultivation zone	0.3566	0.2618	0.3396	0.4290	0.4522
China	0.3565	0.3237	0.3132	0.2458	0.1761

(3) The difference of factor interaction influence in each sub-district is significant, which becomes the key to improving the transformation efficiency of red tourism resources. From the results of the interaction influence of each dominant factor on the transformation efficiency of China's red tourism resources in the subregion, we get: that endogenous capacity and the external environment factor interaction are the most important ways to influence the transformation efficiency of tourism resources in different subregions, but presenting two-factor combination of the type of influence of the differentiation and the combination of the influence of the diversification of the mode of geographic characteristics (Table 4). The influence of the dominant factors are all high or low values of the regional internal and external interaction influence mode is simple but the combination type difference is significant. Among them, the six dominant factor influences in the synergistic zone of red tourism resources input and output efficiency are all high values and E2 and E9 (QE2 ∩ E9 is 0.1586) become the most significant types of interaction influences; the system supply leadership and scientific and technological innovation reliance become the advantages of promoting the transformation efficiency of red tourism resources represented by Beijing, and also the focus of the future polarization, which is the recommended paradigm for the high-quality development of red tourism. The influence of each dominant factor in the red tourism resources comprehensive capacity enhancement area is low, and E3 and E7 (QE3 ∩ E7 is 0.1498) become the types with the most significant interaction effects; the exploration of the linkage mechanism between tourism human resources cultivation and economic level is the focus of the concern for promoting the transformation efficiency of red tourism resources represented by Jiangxi. The regional interaction influence pattern and combination type of the influence differentiation of the dominant factors are complicated. Among them, the endogenous ability factor interaction of the red tourism industry comprehensive quality enhancement and efficiency area is significant, and the most prominent types of performance are E3 and E4 (QE3 ∩ E4 is 0.1279), which become the advantages of the region; while the shortcomings of the interaction influence of the external environment are E7 and E9 (QE7 ∩ E9 is 0.1005). Therefore, the complementary paths of appropriate evacuation of endogenous force energy, improvement of policy and institutional supply, and capital investment are the focuses of realizing the transformation efficiency upgrading of red tourism resources represented by Hunan. The external environment interactions in the red tourism resource efficiency transformation and upgrading area are dominated by E6 and E9 (QE6 ∩ E9 is 0.1213), but the interaction influence between E4, E3, and E7 is significantly smaller. Therefore, the implantation mode of enhancing endogenous capacity and the empowerment mode of the external environment becomes the key to promoting the growth of transformational efficiency of red tourism resources in the region. The internal and external interactions among E4, E6 and E9 in the comprehensive effectiveness cultivation area of red tourism resources are obviously in a weak position (QE4 ∩ E9, QE6 ∩ E9, QE4 ∩ E6 are 0.0981, 0.0936 and 0.0997, respectively), and the vitality of polarized market players, the enhancement of transportation accessibility and the policy guidance have become the key means to break the trap of the conversion efficiency of the red tourism resources in this region.

**Table 4:** Results of interactive detection on spatial differences of the transformation efficiency of red tourism resources in China in 2022.

Region	Q-value	Dominant Impact Factor				
		E3	E4	E5	E8	E9
China	E3	0.0798*				
	E4	0.0182BE	0.0803NE			
	E5	0.0843*	0.0579NE	0.0910*	0.0498*	
	E8	0.0705NE	0.0104NE	0.0520*	0.0398*	0.0963*
	E9	0.0853NE	0.0807*	0.0496	0.0814*	0.0398*
Production efficiency synergy zone	E3	0.0860*				
	E4	0.0847*	0.0872BE	0.0074*		
	E5	0.0298BE	0.0412NE	0.0023BE	0.0036*	
	E8	0.0577*	0.0714*	0.0312NE	0.0814*	0.0469NE
	E9	0.0288NE	0.0573BE	0.0694*	0.0472NE	0.0379*
Improving quality and efficiency zone	E3	0.0195*				
	E4	0.0473*	0.0295*	0.0323*		
	E5	0.0382NE	0.0064BE	0.0698*	0.09404BE	
	E8	0.0817NE	0.0078BE	0.0680*	0.0027*	0.0261*
	E9	0.0807BE	0.0447NE	0.0555BE	0.0495*	0.0448*
Upgrading efficiency conversion zone	E3	0.0761*				
	E4	0.0545*	0.0331*	0.0591*		
	E5	0.0918BE	0.0779*	0.0824BE	0.0995*	
	E8	0.02692	0.0850BE	0.0916*	0.0688*	0.0511*
	E9	0.0616NE	0.0113*	0.0111NE	0.0973BE	0.0190NE
Improvement production capacity zone	E3	0.0791*				
	E4	0.0636*	0.0858BE	0.0256*		
	E5	0.0985*	0.0928NE	0.0056*	0.04356*	
	E8	0.02103NE	0.08198NE	0.0281BE	0.0023*	0.0079*
	E9	0.0936BE	0.0037*	0.0249NE	0.0452*	0.0723NE
Effectiveness cultivation zone	E3	0.01597*				
	E4	0.0198*	0.0998NE	0.0970*		
	E5	0.0772BE	0.0743*	0.0284*	0.00587BE	
	E8	0.0813NE	0.0696BE	0.0921NE	0.0805*	0.0511*
	E9	0.0422BE	0.02734*	0.0326*	0.0692NE	0.0444NE
*VT*_*E*_		0.7966	0.3184	0.1108	0.8624	0.5383

***, **, and * represent significant at 1%, 5%, and 10% significance levels, respectively.

## Discussion and conclusion

### Discussion

The study addressed a limitation in the existing literature by introducing a modified DEA model to measure the conversion efficiency of input elements within red tourism resources, overcoming the previous inability to determine this efficiency. Geographical perspectives such as spatial correlation and spatial spillover, spatial autocorrelation, and geographic detector models were employed to explore the influencing factors and mechanisms behind the conversion efficiency of red tourism resources. These results are consistent with previous research^37^.

At the same time, compared with the comprehensive efficiency of tourism resources put into operation evaluated by the traditional DEA model, the application of the modified DEA model to evaluate the transformation efficiency of red tourism resources can decompose the comprehensive efficiency of the effective decision-making unit of non-variable returns to scale, and realize the extraction of single factor efficiency of single input and output variables. The inherent “black box” problem of the traditional DEA model is avoided, and the bias and irrationality of the overall efficiency evaluation of the model are compensated. As the carrier of tourism activities, red tourism resources are unique thematic, historical, and cultural resources integrating the values and functions of revolutionary traditional education, advanced cultural dissemination, and tourism economic expansion. Evaluating the production and conversion efficiency of red tourism resources helps accurately grasp the connotation of red tourism resources, objectively evaluating their value and guiding the sustainable development of red tourism. Therefore, in developing red tourism resources, precise policies should be taken according to the current characteristics and development dynamics of red tourism resources in various regions, and differentiated resource transformation ideas should be put forward for red tourism resources at different levels and in different regions. For the regions with high conversion efficiency of red tourism resources, the redundancy of financial and human resources should be reduced as much as possible based on satisfying the technological innovation investment. In areas with low conversion efficiency of red tourism resources, investment in resource development and utilization should be increased, and attention should be paid to the optimal allocation of resources and the improvement of scale efficiency.

However, owing to space limitations, this study still has several shortcomings: first, the selection of 300 typical red tourism resource attractions in China was primarily constrained by data availability. This potentially biases the overall efficiency inference of China’s red tourism resources owing to their larger scale. Second, owing to data limitations, this study did not consider the efficiency of tourism resource transformation under environmental constraints, which warrants further investigation.

### Conclusion

This study evaluates the resource transformation efficiency of 300 red tourist attractions in China in 2022 and its interaction influence mechanism by using a modified DEA model, spatial autocorrelation method, and geodetector model, and obtains the following conclusions:

1. China’s red tourism resources commissioning efficiency deviates from the frontier surface, showing the spatial characteristics of gradually decreasing from east to west. 2022 China's red tourism resources commissioning total efficiency average value of 0.720, the commissioning efficiency, market efficiency, and resource endowment efficiency of different resource units and provinces vary greatly, and on the whole, shows the spatial change trend of gradually decreasing from east to west. The low technical innovation capacity and the poor allocation of tourism resources are both the current situation of the production efficiency of China's red tourism resources and an important factor constraining the improvement of production efficiency.
2. The conversion efficiency of the western region is 0.407 lower than that of the eastern and southern regions, but its composition of conversion efficiency is different, that is, the conversion efficiency of social effects is high but the conversion efficiency of public opinion effects is low. Owing to the imperfect red tourism market in the western region, imperfect transportation network conditions, and inadequate promotion of red tourism, the spillover effects of tourists’ “words and deeds” and tourism enterprises’ “advanced experience” in neighboring areas are insufficient, hindering the transformation of public opinion benefits.
3. The quality of human capital, the scale of the tourism market, the structure of the tourism industry, the level of urbanization, and the strength of policy support are identified as the dominant factors affecting the spatial differentiation of the transformation efficiency of red tourism resources. The influence of the dominant influencing factors varied significantly, and the comprehensive influence of the external environment became the main source of influence.
4. The interaction influence of two-factor is stronger than that of one-factor and dominated by nonlinearly enhanced interaction type. The factor interaction influences of the five types of regions of China's red tourism resource transformation efficiency all show nonlinear enhancement and two-factor enhancement, and there is no independent and weakened relationship. The two-factor interaction influence showed differences in internal and external types, and the cumulative cyclic effect of the endogenous ability factor group was more significant. The difference of factor interaction influence in each sub-district is significant, which becomes the key to improving the transformation efficiency of red tourism resources.

## Data Availability

The datasets generated during and/or analysed during the current study are available from the corresponding author upon reasonable request.
